# Dose health education on dementia prevention have more effects on community residents when a community physician/nurse leads it? A cross-sectional study

**DOI:** 10.3389/fpubh.2023.1101913

**Published:** 2023-05-03

**Authors:** De Gong, Yan Peng, Xiao Liu, Jinying Zhang, Menghui Deng, Tiantian Yang, Yanni Yang

**Affiliations:** School of Nursing, Third Military Medical University/Army Medical University, Chongqing, China

**Keywords:** dementia prevention, health education, knowledge, motivation, lifestyle

## Abstract

**Background:**

Dementia is a growing public health concern worldwide. Community residents still have limited knowledge about dementia prevention, although many sources are accessible for individuals to acquire knowledge.

**Methods:**

A questionnaire-based survey was conducted in five communities in Chongqing, China, between March 2021 and February 2022. Participants were divided into three groups according to the dementia-related education they received: physician/nurse-led, mass media, and no relevant education. Covariance analysis was performed to determine the differences among the three groups in knowledge, motivation, and lifestyle, with the covariate of MoCA scores (education-adjusted).

**Results:**

Of the 221 participants, 18 (8.1%) received physician/nurse-led education, 101 (45.7%) received only mass media education, and 102 (46.2%) did not receive any relevant education regarding dementia prevention. Participants who only received mass media education had a higher level of education (*t* = 5.567, *p* = 0.004) and cognitive function (*t* = 13.978, *p* < 0.001). The analysis of covariance showed that compared with participants who received no relevant education, those who received physician/nurse-led education had higher levels of knowledge, perceived benefits, and better lifestyle, and those who received mass media education had lower perceived barriers; however, higher levels of cues to action, general health motivation, self-efficacy, and lifestyle (all *p* < 0.05).

**Conclusion:**

The popularization of dementia-related education was not ideal for communities. Physician/nurse-led education plays a vital role in providing knowledge and promoting lifestyles for dementia prevention, but may not motivate community residents. Mass media education may help encourage residents and promote their lifestyles.

## 1. Introduction

Dementia is a health problem for both affected individuals and caregivers, and is a growing public health problem worldwide. The prevalence of dementia is increasing rapidly, with nearly 10 million new cases reported each year worldwide with the aging of the global population (1). Although there is no curative treatment for dementia, increasing evidence has proven that dementia can be prevented. Lifestyle factors may influence the onset or progression of dementia, and approximately 40% of dementia is attributable to a combination of 12 risk factors, including education, psychological factors, chronic disease, and smoking (2). Recently, three large randomized controlled trials on dementia prevention also proved that multidomain lifestyle interventions could be helpful in preventing cognitive impairment (3–5). Therefore, new evidence of optimal strategies to improve the knowledge and ability of community residents to prevent dementia is worth exploring.

The public awareness of dementia prevention remains limited (6), particularly in undeveloped regions. A survey (7) of an international sample found that individuals from Africa and India had lower performance on the dementia knowledge assessment scale (DKAS) than other developed regions, such as the United States and Canada. As dementia-related symptoms often occur in older adults, dementia is often regarded as an inevitable result of normal aging. According to the World Alzheimer Report 2019 (8), two in three people think that dementia is caused by normal aging, one in four people think that dementia is not preventable, and up to 95% of the public believe that they will eventually develop dementia. These misconceptions about dementia may not only increase the negative psychological reactions of people, such as fear and anxiety, but also impede active strategies for dementia prevention and coping among community residents.

Zheng et al. (9) found that 74–90% of 3,338 Chinese participants were aware of the impact of lifestyle on the development of dementia. Among 604 young Australian adults, it was reported approximately 70% participants demonstrated some understanding of dementia, and approximately half agreed with nine established dementia risk factors (10). The Alzheimer’s Association, in partnership with the Center for Disease Control and Prevention, has recently introduced two initiatives to maintain brain health. One of the recommendations was to “identify and promote culturally appropriate strategies designed to increase public awareness of dementia, including Alzheimer’s disease, to reduce conflicting messages, stigma, and promote early diagnosis (11).” Therefore, it is necessary to promote science popularization by spreading awareness of dementia prevention and ultimately improving the overall health literacy about dementia prevention among community residents.

Many resources are accessible to individuals for acquiring relevant knowledge about dementia prevention. In particular, medical staff play an important role in disseminating information. Many studies have advocated and proven the effectiveness of nurse/physician-led education for patients or community residents, which could effectively improve individuals’ ability to prevent and manage dementia (12–16). In addition to medical staff, individuals could also gain knowledge of diseases from mass media, such as books, television, and the Internet. Regarding dementia awareness, a mass media observation project reported that an increase in awareness of dementia could be due to high profile or celebrity exposure and a range of representations in movies, television, and books (17). Moreover, mass media sources have widely complemented and encouraged specific educational interventions in most studies (18–20). However, in addition to research-based educational interventions, data on the availability and accessibility of dementia-related information for the community residents remain unclear. Therefore, investigating education sources that are effective in improving individuals’ knowledge, motivation, and behaviors regarding dementia prevention is vital.

To identify the different effects of dementia prevention awareness provided by physician/nurse and mass media, this study involved community residents and surveyed the sources from which they acquired awareness of dementia prevention. Middle-aged and older adults were recruited, considering of their higher dementia preventive potential. Although dementia mostly occurs in old age, cognitive decline begins in middle age (21). The effects of the two types of awareness sources on residents’ knowledge, motivation, and behaviors toward dementia prevention were investigated. The findings of this study will provide evidence and support for establishing effective methods for providing awareness of dementia prevention in the future.

## 2. Materials and methods

### 2.1. Study setting

This was a cross-sectional study. Between March 2021 and February 2022, residents aged ≥45 years from five communities in Chongqing, China was invited to participate in this study. Community residents were recruited at a large community health service center that provided health services to over 56,000 permanent residents in these five communities. Each year, the community health service center provides routine training about health education, and all community physicians and nurses in these five communities have the same access to these training.

### 2.2. Participants

Middle-aged and older adults were recruited by convenience sampling from five communities in Chongqing, China. Participants were recruited face-to-face when they voluntarily visited community health services. About 20 yuan or an equivalent gift would be provided with appreciation for each participant who completed all questionnaires and scales. The inclusion criteria were as follows: (1) age ≥ 45 years, (2) provision of informed consent, and (3) ability to read and fill in the required questionnaire independently (or with the help of the researchers). The exclusion criteria were as follows: (1) clear diagnosis of various types of dementia and (2) severe visual or hearing impairment and unable to communicate effectively.

### 2.3. Data collection

This study was approved by the Medical Ethics Committee of Army Medical University (2021 NO. 23-02). Between March 2021 and February 2022, residents of five communities were invited to participate in this study, and the study objectives and main points were explained. To avoid bias in data collection, all data collectors received uniform training before the study. The training and data collection tools were designed by the study designer, who is a professor of geriatric care. All data were self-reported by the participants. Each participant took about 30 min to complete all questionnaires and scales. A second interview was conducted with respondents with incomplete questionnaires. Residents who could not be contacted were excluded from this study. Finally, 232 community residents were invited, of whom 221 provided informed consent and completed the survey.

### 2.4. Measures

#### 2.4.1. Demographic and dementia-related characteristics questionnaire

The questionnaire solicited the participants’ demographic characteristics: sex, age, years of education, work status, marital status, living status, family history, history of contact with dementia patients, and belief in dementia prevention (Can dementia be prevented?). An additional question was also added to the questionnaire, “Have you received any dementia-related education,” answering options included “yes” and “no”. Option “yes” included the following information sources: television programs, Internet, community physician/nurse, books and newspaper, community health lectures, and others with specific descriptions.

#### 2.4.2. Montreal cognitive assessment

Montreal cognitive assessment (MoCA) was developed by Nasreddine et al. (22) to measure an individual’s cognitive function in seven cognitive areas: orientation, language, working memory, delayed memory, executive function, and visuospatial ability. The total MoCA score was 30, with a higher score indicating a higher level of cognitive function. Considering the impact of education level on cognitive function, the MoCA score can be adjusted for years of education. One point is added to the original MoCA score if an individual is educated for less than 12 years. The Beijing version (23) of the MoCA, which has been modified from a cultural and linguistic perspective, was used in this study. In this study, the Beijing version of MoCA was employed through face-to-face interviewing, which took approximately 10 min for each participant. The sensitivity of the Beijing version of the MoCA was 83.8% for all cognitive impairments, 80.5% for mild cognitive impairments, and 96.9% for dementia, and the specificity for identifying cognitively normal individuals was 82.5%. The use of MoCA Scale Beijing version for cognitive function screening is a recommended item in “Consensus of Chinese memory physical examination experts” (24). The screening was carried out as a physical examination item in the community health service center where this study was conducted and specialized training in cognitive screening was implemented regularly.

#### 2.4.3. Risks and health promotion subscale of dementia knowledge assessment scale

The DKAS (25) is a 25-item scale developed by Annear et al. in 2015 to measure the level of dementia-related knowledge in the general population. The DKAS was completed by participants. It consists of four subscales: causes and characteristics (seven items), communication and behavior (six items), care considerations (six items), and risks and health promotion (six items). This study focused on participants’ knowledge related to dementia prevention; therefore, the subscale of risks and health promotion was employed in this study. There are five potential responses: “false, probably false, probably true, true, and I do not know.” In true descriptions, for the true, probably true, and other answers, two, one, and zero points, respectively, were provided. In false descriptions, for the false, probably false, and other answers, two, one, and zero points, respectively, were provided. The total score on the risks and health promotion subscale ranged from 0 to 12, with a higher score indicating a higher level of related knowledge. The DKAS has been verified in China, in which the Cronbach’s alpha value of the Risks and Health Promotion subscale was 0.83.

#### 2.4.4. Motivation to change lifestyle and health behaviors for dementia risk reduction

The Motivation to change lifestyle and health behaviors for dementia risk reduction (MCLHB-DRR) is a 27-item scale developed in Australia to measure beliefs and attitudes toward dementia and dementia risk reduction (26). The MCLHB-DRR was completed by participants. There are seven subscales: perceived susceptibility (four items), perceived severity (five items), perceived benefits (four items), perceived barriers (four items), cues to action (four items), general health motivation (four items), and self-efficacy (two items). All items were rated on a 5-point Likert scale ranging from 1 (strongly disagree) to 5 (strongly agree). This scale is scored on the subscales, and a higher score indicates a higher level of motivation to change lifestyles. The MCLHB-DRR was validated in Australia (26), Turkey (27) and Netherlands (28). The scale was cross-culturally adapted to the Chinese version using Bristlin’s forward and backward translation protocol (29) in our previous work (30). The Chinese version showed a Kaiser–Meyer–Olkin value of 0.74, and Cronbach’s alpha for the scale was 0.76.

#### 2.4.5. Lifestyle for dementia risk reduction

The Lifestyle for dementia risk reduction (LDRR) (31) was designed in our previous studies to assess an individual’s lifestyle related to dementia risk reduction based on a health promotion model (32) and literature review for dementia risk reduction. Participants would fill in the LDRR according to their living habits during the last month. This is a 32-item scale with eight dimensions: health responsibilities, physical activity, mental activity, nutrition, tobacco and alcohol habits, interpersonal relationships, stress management, and spiritual growth. Responses were measured on a 4-point Likert scale from 1 (never) to 4 (always). The total score ranges from 32 to 128, with a higher score indicating a healthier lifestyle to reduce the risk of dementia. This finding has been validated in the Chinese population. Exploratory factor analysis showed acceptable results with the cumulative variance contribution rate 60.19% and the factor loadings ranged from 0.40 to 0.87. The fitness indices of the confirmatory factor analysis also reached acceptable levels. Moreover, the Cronbach’s alpha of this scale was 0.86, and its test–retest reliability was 0.86.

### 2.5. Statistical analysis

SPSS software (version 25.0; IBM Corp., Armonk, NY, USA) was used for the statistical analysis, and statistical significance was set at *p* ≤ 0.05. G*Power software (version 3.1.9.7) was used to test the statistical power (1-β) with sample size (33), and the effect size was set as 0.25 with *α* = 0.05. Frequency and percentage were used to describe classification variables, including sex, work status, marital status, living status, family history, and history of contact with dementia patients. Mean and standard deviation (SD) were used to describe the continuous variables, including age, years of education, MoCA scores (education-adjusted), knowledge, motivation, and lifestyle. Participants were divided into three groups according to the dementia-related education they received: physician/nurse-led, mass media, and no relevant education. Chi-square tests were performed to determine the differences among the three groups in the classification variables, and the Fisher’s exact test would be employed when the minimum expected count was less than five. One-way ANOVA tests were used to determine the differences among the three groups in age, years of education, and MoCA scores (education-adjusted). The least significant difference test was used for *post hoc* multiple comparisons. Finally, covariance analysis was used to determine the differences among the three groups in knowledge, motivation, and lifestyle, with the covariate of the MoCA scores (education-adjusted). In this research, data from participants who answered the complete survey were included; no imputations of missing data were made.

## 3. Results

### 3.1. Demographic characteristics

A total of 221 participants were included in this study, and the statistical power (1-β) calculated with this sample size was 0.92. Mean age of these participants was 66.84 (standard deviation, SD: ±7.71), and their mean education years was 8.45 (SD: ±3.83). Most participants were women (142, 64.3%), retired (189, 85.5%), married (176, 79.6%), and considered dementia as preventable (174, 78.7%). Furthermore, a few participants lived with patients with dementia (23, 10.4%), and a few had a family history (21, 9.5%), whereas approximately half (108, 48.9%) had been in contact with dementia patients before. The detailed data are presented in Table 1.

**Table 1:** Demographic characteristics and the differences among the participants received different education (*N* = 221).

	Total (*n* = 221)	Physician/nurse-led education (*n =* 18)①	Mass education (*n =* 101)②	No related education (*n =* 102)③	*X*^2^/*F*/*t*^†^	*p*
Sex, *n*(%)						
Male	79(35.7)	9(50.0)	33(36.3)	37(36.3)	2.020	0.364
Female	142(64.3)	9(50.0)	68(63.7)	65(63.7)		
Age, mean ± SD	66.84 ± 7.71	69.22 ± 5.84	66.84 ± 7.58	66.42 ± 8.09	1.011	0.366
Education years, mean ± SD	8.45 ± 3.83	7.67 ± 2.59	9.39 ± 3.66	7.67 ± 3.99	5.767	0.004
					②③3.268	0.001
Work status, *n*(%)						
Retired	189(85.5)	15(83.3)	91(90.1)	83(81.4)	3.343	0.202
On-the-job	32(14.5)	3(16.7)	10(9.9)	19(18.6)		
Marital status, *n*(%)						
Married	176(79.6)	13(72.2)	81(80.4)	82(80.2)	0.834	0.740
Unmarried, divorced and widowed	45(20.4)	5(27.8)	20(19.6)	20(19.8)		
Living status, *n*(%)						
Living along	23(10.4)	2(11.1)	12(11.9)	9(8.8)	0.658	0.705
Living with others	198(89.6)	16(88.9)	89(88.1)	93(91.3)		
Family history of dementia, *n*(%)						
Yes	21(9.5)	2(11.1)	8(7.9)	11(10.8)	0.747	0.727
No	200(90.5)	16(88.9)	93(92.1)	91(89.2)		
History of contact with dementia patients, *n*(%)						
Yes	108(48.9)	12(66.7)	48(48.0)	48(47.7)	2.489	0.309
No	113(51.1)	6(33.3)	53(52.0)	54(52.3)		
Can dementia be prevented? *n*(%)						
Yes	174(78.7)	16(88.9)	87(86.1)	71(69.6)	9.052	0.009
No	47(21.3)	2(11.1)	14(13.9)	31(30.4)	②③8.038	0.005
MoCA^‡^ scores	23.23 ± 4.49	21.50 ± 6.62	24.87 ± 2.97	21.91 ± 4.77	13.978	<0.001
					①②3.107	0.002
					②③4.966	<0.001

† Chi-square tests were performed to determine the differences for the classification variables. One-way ANOVA tests were used for the continuous variables, and the least significant difference test was used for *post hoc* multiple comparisons.

‡ MoCA, Montreal cognitive assessment.

### 3.2. Sources of dementia-related education

**Figure 1:**
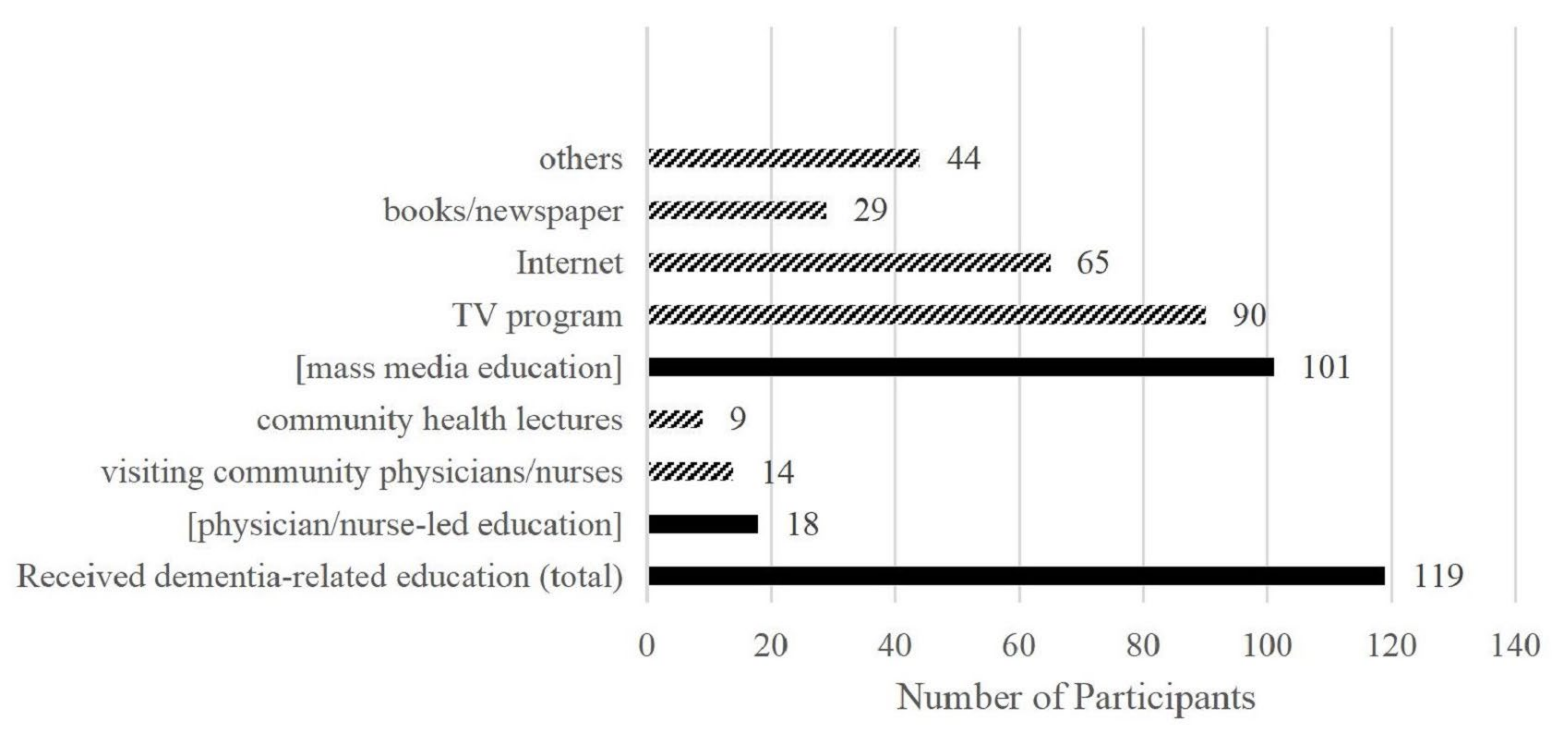
Number of participants receiving dementia-related education from different sources.

A total of 119 participants had previously received dementia-related education and 102 participants had not received relevant education. Regarding the 119 participants, most were educated through television programs (90, 75.6%), the Internet (65, 54.6%), and others (44, 37.0%). The “others” here is mainly relatives/friends’ information, with some movie, radio and etc. Only a few participants were educated by visiting community physician/nurse (14, 11.8%) and community health lectures (9, 7.6%). Some participants were educated through more than one path. After statistical integration, 18 (8.1%) participants received physician/nurse-led education (including visiting community physician/nurse and community health lectures), and 101 (45.7%) received only mass media education (including television programs, the Internet, books, newspapers, and others). The detailed data are presented in Figure 1.

### 3.3. Demographic differences among participants who were educated differently

As shown in Table 1, there were no statistical differences in most demographic characteristics among the three groups, except education years and beliefs in dementia prevention. Compared with participants who received no dementia-related education, those who received mass media education had more years of education (*t* = 3.268, *p* = 0.001), and more of them believed dementia could be prevented (*X*^2^ = 8.038, *p* = 0.005). Participants who received mass media education also had higher MoCA scores than those of the other groups (*t*_1_ = 3.107, *p*_1_ = 0.002; *t*_2_ = 4.966, *p*_2_ < 0.001).

### 3.4. Analysis of covariance on the knowledge, motivation, and lifestyle scores

There were no statistical differences in demographic characteristics, except years of education, among the three education groups. Considering the differences in years of education, MoCA scores among the three groups, and the potential influence of education level and cognitive function on individuals’ knowledge acquisition, motivation generation, and behavior style, education-adjusted MoCA score was employed as a covariate, which showed different levels in the three education groups (*F* = 13.655, *p* < 0.001). Analysis of covariance showed that only participants who received physician/nurse-led education had a higher knowledge of dementia prevention than those who received no related education (*t* = 2.375, *p* = 0.018). The results showed that compared with participants who received no related education, those who received physician/nurse-led education had higher levels of perceived benefits (*t* = 2.091, *p* = 0.038), and those who received mass media education had lower levels of perceived barriers (*t* = 2.183, *p* = 0.030) higher levels of cues to action (*t* = 2.131, *p* = 0.034), general health motivation (*t* = 1.982, *p* = 0.049), and self-efficacy (*t* = 2.076, *p* = 0.039). Regarding lifestyle scores, participants who received physician/nurse-led education (*t* = 2.520, *p* = 0.012) and mass media education (*t* = 2.347, *p* = 0.020) had better lifestyles than those who received no relevant education. The detailed data are presented in Table 2.

**Table 2:** Analysis of covariance on the knowledge, motivation and lifestyle scores among participants who received different types of education (*N* = 221).

	Uncorrected			Corrected^†^			*F*/*t*	*p*
	Physician/nurse-led education (*n =* 18)	Mass media education (*n =* 101)	No related education (*n =* 102)	Physician/nurse-led education (*n =* 18)①	Mass media education (*n =* 101)②	No related education (*n =* 102)③		
MoCA scores (education-adjusted)	22.39 ± 6.52	25.58 ± 2.86	22.70 ± 4.71	-	-	-	13.655	<0.001
Knowledge	8.28 ± 2.08	7.77 ± 2.42	6.80 ± 2.79	8.52 ± 0.59	7.53 ± 0.26	7.00 ± 0.25	3.189	0.043
							①③2.375	0.018
Motivation								
perceived susceptibility	9.72 ± 4.07	9.89 ± 4.04	9.83 ± 4.53	9.41 ± 1.00	10.20 ± 0.43	9.58 ± 0.43	0.575	0.564
perceived severity	16.78 ± 5.04	17.77 ± 5.77	17.88 ± 5.41	16.24 ± 1.28	18.30 ± 0.55	17.45 ± 0.55	1.276	0.281
perceived benefits	19.56 ± 0.78	18.86 ± 2.03	18.44 ± 2.29	19.59 ± 0.50	18.83 ± 0.22	18.47 ± 0.21	2.402	0.093
							①③2.091	0.038
perceived barriers	8.61 ± 5.48	7.60 ± 4.26	9.38 ± 5.07	8.47 ± 1.13	7.74 ± 0.49	9.27 ± 0.48	2.388	0.094
							②③2.183	0.030
cues to action	13.78 ± 4.33	13.71 ± 4.49	12.26 ± 4.52	13.79 ± 1.07	13.70 ± 0.46	12.28 ± 0.46	2.592	0.077
							②③2.131	0.034
general health motivation	19.39 ± 1.30	18.88 ± 1.99	18.27 ± 2.62	19.36 ± 0.54	18.91 ± 0.23	18.25 ± 0.23	3.028	0.050
							②③1.982	0.049
self-efficacy	9.39 ± 0.78	9.27 ± 1.54	8.66 ± 1.95	9.44 ± 0.40	9.22 ± 0.18	8.70 ± 0.17	2.899	0.057
							②③2.076	0.039
Lifestyle	94.17 ± 11.40	92.02 ± 12.73	86.63 ± 11.38	94.86 ± 2.82	91.32 ± 1.22	87.19 ± 1.21	4.737	0.010
							①③2.520	0.012
							②③2.347	0.020

† Corrected for the education-adjusted Montreal cognitive assessment (MoCA) score.

## 4. Discussion

The results indicated that there were significant differences among participants who received different types of education: (1) approximately half of the participants received dementia-related education from mass media and medical staff, whereas only a few received education from physicians or nurses; (2) participants who only received mass media education had higher levels of education and MoCA scores; and (3) after adjusting the MoCA scores (education-adjusted), compared with participants who received no relevant education, those who received physician/nurse-led education had higher levels of dementia-related knowledge, perceived benefits, and a better lifestyle, whereas those who received mass media education had higher levels of perceived barriers, cues to action, general health motivation, self-efficacy, and a better lifestyle.

The popularization of dementia-related education, especially physician/nurse-led education, was not ideal in this study. Although several studies have reported knowledge and awareness of dementia in the public (6, 34), only a few studies have explored the popularity rate of dementia-related education among community residents. Zhang et al. (35) reported that dementia-related education was rarely provided in local Chinese communities, and that dementia-related education in communities is mostly the independent and spontaneous behavior of community physician/nurse. Moreover, community residents may not be willing to seek medical help unless they experience obvious symptoms or the threat of dementia. However, very subtle cognitive alterations may exist and be detectable before meeting the criteria for mild cognitive impairment, which requires community physician/nurse to act as early as possible (36).

In addition, the results indicated that people with better education and cognitive function preferred mass media education. Their knowledge and cognitive abilities may have led them to seek health-related information and they were willing to access relevant knowledge through mass media sources. However, approximately half of the participants had received no relevant education and had lower education and cognitive function, which means that it may be more difficult for them to recognize their cognitive problems, and they may even have a degree of cognitive impairment. They lacked awareness and the ability to seek help actively. Therefore, community physician/nurse should focus more on community residents with low levels of education and cognitive-function to provide targeted education and support. Considering the differences in years of education and MoCA scores among the three groups, and the potential influence of education level and cognitive function on individuals’ knowledge acquisition, motivation generation, and behavior style, the education-based cognitive function was adjusted. Detailed results and discussions of covariance analysis were shown as follows:

Participants who received physician/nurse-led education had better dementia-related knowledge than those who received no relevant education, indicating a potential relationship between physician/nurse-led education and better dementia-related knowledge. Sandra et al. (37) and Wiese et al. (38) confirmed the positive effect of physician/nurse-led education on dementia among different populations. Regarding chronic diseases such as diabetes, hypertension, and hyperlipidemia, the physician/nurse-led education model is widely employed to promote patients’ self-management of lifestyle, medicine intake, and monitoring (39). Therefore, it may also be worth to employ physician/nurse-led education on dementia prevention in communities. Meanwhile, some understanding of dementia may simultaneously encourage community residents to seek physician/nurse-led education. Kwak et al. found that knowledge could affect the healthcare-seeking behaviors of older residents with dementia (40). From another perspective, this may suggest the importance of mass media education on dementia prevention, which can provide some basic, easy knowledge of dementia, to promote individuals’ seeking behaviors in physician/nurse-led education.

Participants who received physician/nurse-led education had higher perceived benefits. In this study, perceived benefits are beliefs about the positive features or advantages of a recommended action to reduce the risk of dementia (41). Compared to mass media education, physician/nurse inevitably bring about subjective perspectives in educational practice. From the perspective of medical ethics, physician/nurse are more inclined to show optimism, encourage, and provide positive information, which may enhance the perceived benefits among community residents. However, there was no significant difference in other dimensions between participants who received physician/nurse-led education and those who received no related education, which may suggest that although physicians and nurses have provided more professional knowledge in actual health education, they may motivate individuals’ behavior less. In a qualitative study among primary health workers (42), it was found that they were unable to help a person make lifestyle changes unless they were motivated to do so. Some effective methods have been reported to improve motivation, such as motivation interviewing, which has been widely employed in interventions for disease self-management [hypertension (43), diabetes (44), and obesity (45)]. Therefore, it is necessary to improve the knowledge and capacity of health workers for motivational interventions.

This study also found that participants who received mass media education had fewer perceived barriers more cues to action, general health motivation, and self-efficacy, and more believed that dementia could be prevented. However, these significant differences were not found between participants who received physician/nurse-led education and those who received no relevant education, suggesting that mass media education has a more positive effect on enhancing individuals’ behavioral motivation. In contrast, community physician/nurse may focus more on knowledge transmission rather than motivation stimulation in health education. With the development of information technology, people can access various aspects of disease knowledge through different media to address the barriers they encounter in their living environment. Most of the published media materials are carefully designed, which may provide more systematic, understandable, and operational, suggestions that increase cues to action, general health motivation, and self-efficacy in people (44, 46). It should also be noted that these participants (mass media education group) were already highly motivated and needed suitable guidance from community physician/nurse regarding a lifestyle for brain health.

This study found that participants who received physician/nurse-led education and mass media education had better lifestyles. Partially, a better lifestyle may suggest better health literacy, which indicates that people may focus more on health and be more likely to seek related information. In addition, this study also suggests that education has positive effects on promoting a healthy lifestyle. With increasing evidence supporting the protective mechanisms of lifestyle on dementia, it is necessary to improve the knowledge and educational ability of community physician/nurse on lifestyle for brain health, simultaneously, to enrich the resources of mass education and then help more residents at risk of dementia or with preventive potential of dementia learn more about dementia prevention.

This study had several limitations. First, the sample size and the sampling method in this study were limited, because the MoCA test, which required face-to-face interview, was time-consuming, and the COVID-19 pandemic also increased the difficulty of data collection. It’s worth noting that the *post hoc* test of the sample size in this study shows a good result (1-β = 0.92), which may support the credibility of the final results. Second, the number of the participants who received physician/nurse-led education was smaller than other two types, which may partially limit the reliability of the results; however, it was still a true reflection of the healthcare situation in local communities. In the future, more research centers and larger samples should be considered to further investigate dementia education in the general population to provide more evidence for promoting dementia prevention in more community residents.

## 5. Conclusion

This study found that the popularization of dementia-related education was not ideal in communities. Physician/nurse-led education plays an important role in providing knowledge on dementia prevention; however, it may not motivate community residents to a great extent. There may be some correlation between mass media education and a higher level of behavioral motivation, and between all kinds of education and a higher level of lifestyle. Therefore, it is important to strengthen the ability of physician/nurse to provide motivational intervention, constantly enrich the resources and improve the quality of mass media education to improve health education for people at risk of dementia.

## Data availability statement

The original contributions presented in the study are included in the article/supplementary material, further inquiries can be directed to the corresponding author: yangyanni@tmmu.edu.cn.

## Ethics statement

The studies involving human participants were reviewed and approved by the Medical Ethics Committee of Army Medical University. The patients/participants provided their written informed consent to participate in this study.

## Author contributions

YY developed the ideas, administrated the project, provided significant academic guidance on manuscript, and revised the manuscript critically for intellectual content. DG and YP drafted and revised the manuscript. DG was responsible for the data analysis. YP was responsible for the literature review and data collection. XL, JZ, and MD assisted in data collection and revised the manuscript. TY revised the manuscript. All authors contributed to the article and approved the submitted version.

## Funding

This study was supported by a grant from the National Social Science Foundation of China (No. 20BRK039). The funders had no role in the study design, data collection and analysis, decision to publish or preparation of the manuscript.

## Conflict of interest

The authors declare that the research was conducted in the absence of any commercial or financial relationships that could be construed as a potential conflict of interest.

## Publisher’s note

All claims expressed in this article are solely those of the authors and do not necessarily represent those of their affiliated organizations, or those of the publisher, the editors and the reviewers. Any product that may be evaluated in this article, or claim that may be made by its manufacturer, is not guaranteed or endorsed by the publisher.

## Abbreviations

MoCA: Montreal cognitive assessment
DKAS: Dementia knowledge assessment scale
MCLHB-DRR: Motivation to change lifestyle and health behaviors for dementia risk reduction
LDRR: Lifestyle for dementia risk reduction
SD: Standard deviation
ANOVA: Analysis of variance
